# Character strengths as protective factors against behavior problems in early adolescent

**DOI:** 10.1186/s41155-022-00217-z

**Published:** 2022-06-01

**Authors:** Cheng Qin, Xiaotong Cheng, Yuyan Huang, Shuang Xu, Kezhi Liu, Mingyuan Tian, Xiaoyuan Liao, Xinyi Zhou, Bo Xiang, Wei Lei, Jing Chen

**Affiliations:** 1 Department of Psychiatry, The Affiliated Hospital of Southwest Medical University, No. 25 Taiping Street, Jiangyang District, Luzhou, 646000 Sichuan China; 2 Laboratory of Neurological Diseases and Brain Function, The Affiliated Hospital of Southwest Medical University, Luzhou, China; 3 Nuclear Medicine and Molecular Imaging Key Laboratory of Sichuan Province, Luzhou, China; 4 College of Clinical Medicine, Southwest Medical University, Luzhou, China

**Keywords:** Character strengths, Behavior problems, Positive psychology, Early adolescent

## Abstract

Character strengths could effectively prevent negative psychological outcomes in adults. However, there was little research conducted among early adolescents. The present study aimed to explore character strengths that were independently related to fewer behavior problems in early adolescents. In total, 521 early adolescents (mean age 10.92 ± 0.04, range 10–12 years) were recruited from primary schools in Sichuan, China. Character strengths were measured using the Values in Action Inventory of Strengths for Youth (VIA-Youth). Behavior problems were measured using the Conners Parent Symptom Questionnaire (PSQ). The results showed that behavior problems were negatively correlated with character strengths (*r* = −0.14 to −0.3, *p* < 0.05 Bonferroni corrected). Character strengths explained a significant proportion of additional variance (14–22%) in five types of behavior problems after controlling the effect of demographic factors (residence, left-behind experiences, maternal education level). Moreover, several specific character strengths showed an independent contribution (*β* = −0.34 to −0.14 for self-regulation, perseverance, zest, humility, and leadership; *β* = 0.21 to 0.34, for hope; all *p* < 0.05) to behavior problems. Our study revealed that character strengths were protective factors against behavior problems in early adolescents.

**Supplementary Information:**

The online version contains supplementary material available at 10.1186/s41155-022-00217-z.

## Introduction

The character is defined as a family of personality traits that manifests in one’s thoughts, feelings, and actions (Park & Peterson, 2003). For most adolescents during most of the development, the character is far from unitary. The different levels and directional shifts of character between ages may be explained by the relational developmental systems (RDS) theory, which posits that development results from interactive, relational processes between individuals and their contexts that unfold over time and individuals (Overton, 2015). Shubert et al. (2019) applied RDS and the orthogenetic principle to character development and found character structure proceeded from being largely diffuse and global in late childhood to more differentiated across adolescence (Shubert et al., 2019). Specifically, in elementary school, youths often have overly positive views of their competencies and are just beginning to develop a coherent sense of self until early adolescence. Only after they enter middle school, the youths will explore a multitude of possible selves and characteristics (Harter, 2015). Therefore, if a child has opportunities to develop good characters during early adolescence, these characters could bring long-lasting benefits, such as reduced risk-related behaviors (Beets et al., 2009).

The Values in Action (VIA) provides a hierarchical classification of good characters, which includes 24 character strengths and bucketed them into six virtues (Peterson & Seligman, 2004). Virtues are the core characteristics that are proposed to be universally valued across cultures, including wisdom, courage, humanity, justice, temperance, and transcendence. Character strengths are positive personality traits that lead to or exemplify the virtues. For example, by exhibiting strengths such as gratitude and hope, people can express the virtue of transcendence. Two assessments have been developed based on the VIA Classification—the VIA Inventory of Strengths (Peterson & Seligman, 2004) and the VIA Inventory of Strengths for Youth (VIA-Youth) (Park & Peterson, 2006). Both questionnaires are self-report measures of the 24-character strengths, except the former is for adults of 18 years and over, while the VIA-Youth is for youths of 10–17 years.

Character strengths are universally and morally valued traits that contribute to optimal psychological functioning (Park & Peterson, 2006; Peterson & Seligman, 2004). There were empirical evidences that showed that character strengths could promote psychological wellbeing and positive behavior in adults. For example, the character strength of love (Pennebaker, 1990), hope/optimism (Seligman, 2006), gratitude (Emmons & Shelton, 2002), and forgiveness (Harris & Thoresen, 2007) have been empirically and positively linked to good physical health. And self-regulation was predictive of physical fitness and healthy behaviors (Proyer et al., 2013). In adolescents, some empirical evidence suggested that character strengths were related to positive psychological/behavioral outcomes as well. For example, self-regulation, honesty, and leadership were found independently associated with the wellbeing and happiness of high school students (15–18 years old) (Toner et al., 2012). Also, perseverance and hope were associated with both positive classroom behavior and school achievement among primary and secondary school students (Wagner & Ruch, 2015). Additionally, love and hope were positively predictive of life satisfaction among adolescents of 11–14 years (Blanca et al., 2017).

Early adolescence is a sensitive stage that can be easily influenced by the environment (Cambron et al., 2018). Behavior problems that occur at this period could become chronic disorders subsequently (Babore et al., 2016). Behavior problems refer to abnormal psychological behaviors in that the severity and duration exceed the normal range allowed by the corresponding age (Weitzman et al., 2015). According to the Conners Parent Symptom Questionnaire (PSQ), the majority of these problems can be classified into five sets: conduct problems, learning problems, psychosomatic, impulsivity-hyperactivity, and anxiety (Goyette et al., 1978). Among students aged 9–14 in China, 11.72% had at least one of these behavior problems (He et al., 2019), which could further lead to more serious behavioral deficits, emotional issues, or bad performance at school. For example, students with conduct problems had an increased risk of poor psychosocial outcomes, such as depression, drug use, and antisocial behaviors (Bevilacqua et al., 2018). Learning problems were related primarily to poor cognitive self-concept (Gadeyne et al., 2004), while psychosomatic problems were linked to a higher prevalence of regular alcohol use and lack of exercise among adolescents (Norell-Clarke & Hagquist, 2016). Moreover, impulsive-hyperactive behaviors were related to increased aggression and depression (Noda et al., 2013), while hyperactivity usually co-exists with other core features of mania (increased speech, thought disorder, and elevated mood) (Martino et al., 2020). Finally, children with a high level of anxiety could commit more unethical acts due to the feeling of being threatened (Kouchaki & Desai, 2015).

Numerous studies demonstrated that character strengths could effectively prevent negative psychological outcomes in adults. For instance, the character strengths of hope, zest, prudence, love, and forgiveness were negatively associated with distress during the COVID pandemic lockdown (Casali et al., 2021). Character strengths were also associated with low levels of suicidal ideation (Cheng et al., 2020), thus might be acting as protective factors for suicide (Sueki, 2021). Cultivating character strengths through curriculum training remarkably declined depression and anxiety symptoms in undergraduate freshmen (Duan & Bu, 2019). Despite the widespread belief that character strengths are pillars of positive youth development, there were only a few studies that explored the association between character strengths and behavior problems among adolescents. One study revealed that higher levels of wisdom were connected with decreased psychological vulnerability (i.e., vulnerability to the impacts of life events) in adolescents of 14–18 years (Demirci et al., 2019). Another study found that the character strengths of interpersonal and temperance were negatively associated with mental health difficulties and pro-social behavior difficulties among 2061 students of 7–12 years (Shoshani & Shwartz, 2018). These results suggested that certain character strengths may help to reduce behavior problems. However, it is unclear if and how character strengths are related to specific behavior problems. Moreover, it is also unclear if the effect of character strengths is modulated by the demographic factor of the adolescents, given that many of these variables, such as gender, residence, grade, only-child, left-behind experience, and parental education level were found to be associated with behavior problems (Qu et al., 2008; Tao et al., 2007; Yu, 2010).

The present study aimed to explore character strengths that are independently related to fewer behavior problems in early adolescents. Based on previous research, we proposed two hypotheses: (a) higher levels of character strengths are associated with fewer behavior problems in early adolescents, and (b) these associations are not affected by demographic variables.

## Methods

### Participants and data collection

In total, 590 students aged 10 to 13 years in the 4th–6th grade were recruited from primary schools in Chengdu city and Luzhou city (Sichuan, China) from December 2018 to December 2019. Participants who have more than 20% (≥ 30 items) missing items (*n* = 30) and those who always chose one option (*n* = 32) were excluded. Data of 528 respondents were collected and valid (response rate 89%). However, there were only seven 13-year-old respondents. To provide greater consistency with the age period, data of these seven respondents were also excluded. Finally, data of the remaining 521 respondents were analyzed and reported. For the sake of completeness, the results with 13-year-old participants can be found in the supplementary materials (Table S1, S2, S3 and S4); the main findings remain unchanged.

### Measures

Demographic information was collected using a home-designed questionnaire, including gender, age, grade, place of residence (urban vs. rural), parental education level (including 4 levels: primary or lower, junior middle school, senior middle school, and college or higher), only-child, family structure (including 3 levels: two-parent family, multigenerational family, and other family structure), and left-behind experience (yes/no, defined as at least one of the parents cannot accompany the students for more than half a year).

#### Values in Action Inventory of Strengths for Youth (VIA-Youth)

VIA-Youth is a 96-item self-report questionnaire on character strength (Park & Peterson, 2006). The Chinese short version VIA-Youth was used in this study (The VIA Institute on Character, https://www.viacharacter.org/researchers/assessments/via-youth-96). Items are rated on a 5-point Likert scale ranging from 1 (very much unlike me) to 5 (very much like me). The scale provides 24 subscale scores, with 4 items in each subscale, corresponding to the 24-character strengths. The higher scores indicated a higher level of character strengths.

#### Conners Parent Symptom Questionnaire (PSQ)

The PSQ is a parent rating scale of behavior problems in students aged 3–17 years (Goyette et al., 1978). The PSQ has 48 items rated on a 4-point scale (0–3 points). The scale provided measures of five types of behavior problems and an overall hyperactivity index. Higher scores indicated more severe behavior problems. For each behavior problem, a *Z* score was calculated by the average of all items in the corresponding subscale. The Chinese version PSQ has good reliability (Cronbach’s *α* = 0.92) (Su et al., 2001).

### Procedure

The questionnaire was distributed by teachers in the class. The teachers helped in introducing and asking early adolescents and their guardians if they were interested in participating. Children were instructed to fill in VIA-Youth at school, their parents filled in demographic information, and PSQ at home. Both children and their parents were instructed to provide a phone number of the family and the children’s name, age, and gender; this information was used to cross between parents and children. All responses were made through an online survey platform (wjx.cn, https://www.wjx.cn/). No compensation was offered, but a profile of one’s character strengths was available, which was sent to their families through E-mails.

### Data analysis

According to our study of 959 adolescents (contain current sample) (Cheng et al., 2022, under review), four items of the Chinese version VIA-Youth were deleted due to low (< 0.3) item-total correlations (i.e., VIA-5 (*r* = 0.19), VIA-7 (*r* = 0.22), VIA-30 (*r* = 0), and VIA-42 (*r* = − 0.18), belonging to the character strengths of honesty, forgiveness, self-regulation, and prudence respectively). Therefore, we analyzed using the Chinese version VIA-Youth with the remaining 92 items.

Statistical analyses were conducted using SPSS 23.0 (SPSS Inc., Chicago, IL, USA). Independent sample *t*-test and one-way analysis of variance (ANOVA) were used as appropriate to assess the between-subject difference of character strengths and behavior problems on demographic variables. The correlations between character strengths and behavior problems were assessed using Pearson’s correlation. The threshold was set at Bonferroni corrected *p* < 0.05.

Stepwise linear regression was used to identify demographic factors that had independently contribute to the behavior problems. For each model, each behavior problem was entered as the dependent variable separately. Independent variables included gender, grade, only-child, left-behind experiences, residence, parental education level, and family structure.

Hierarchical regression analyses were carried out to test if character strengths could independently predict behavior problems (PSQ scores) in early adolescents while controlling the effects of demographic factors. Predictors were entered in the following order: step 1, extracted demographic factors, and step 2, the 24 traits of character strengths.

## Results

### Sample demographic variables, behavior problems, and character strengths

The sample demographic information was shown in Table 1. The sample contains 521 children of 10–12 years (mean: 10.92±0.04 years), 250 (48%) boys. The majority of the participants were living in urban (59.8%), not only-child (58.6%), without left-behind experiences (60.2%), living in a two-parent family (43.1%), and with parental education level of junior middle school (maternal education level: 41.4%; paternal education level: 44.1%).

**Table 1:** Sample variables and behavior problems

Variables	Options	*n* (%)	M±SD
Gender (boy vs. girl)	Boy	250 (48)	
Age (years)	10	189 (36.3)	
	11	185 (35.5)	
	12	147 (28.2)	
Grade	4	168 (32.2)	
	5	150 (28.8)	
	6	203 (39)	
Residence (urban vs. rural)	Urban	308 (59.8)	
Only-child or not	Only-child	213 (41.4)	
Left-behind experience	Yes	121 (39.8)	
Family structure	Two-parent family	131 (43.1)	
	Multigenerational family	118 (38.8)	
	Others	55 (18.1)	
Maternal education level	Primary or lower	46 (15.1)	
	Junior middle school	126 (41.4)	
	Senior middle school	71 (23.4)	
	College or higher	61 (20.1)	
Paternal education level	Primary or lower	47 (15.5)	
	Junior middle school	134 (44.1)	
	Senior middle school	73 (24)	
	College or higher	50 (16.4)	
Behavior problem	Conduct problems		0.43 ± 0.39
	Learning problems		0.73 ± 0.58
	Psychosomatic		0.18 ± 0.30
	Impulsivity-hyperactivity		0.53 ± 0.54
	Anxiety		0.48 ± 0.46
	Hyperactivity index		0.53 ± 0.45

Descriptive statistics and Cronbach’s *α* coefficients of VIA-Youth were shown in Table 2. Internal consistency of the character strengths ranged from 0.52 (humility) to 0.84 (love of learning). Eighteen of the 24 subscales showed a Cronbach’s *α* coefficient above 0.7. The average inter-item correlations of character strengths were ranged from 0.21 to 0.58, largely within the recommended range of 0.15–0.50 (Clark & Watson, 2016; Swailes & McIntyre-Bhatty, 2002), indicating an acceptable constructive validity.

**Table 2:** Descriptive statistics and Pearson correlations between the 24 character strengths and behavior problems

	M±SD	Skewness	Kurtosis	𝜶	Average inter-item correlation	Conduce problem	Learning problem	Psycho-somatic	Impulsive-hyperactive	Anxiety	Hyperactive index
Appreciation of beauty and excellence	15.38±3.97	−0.56	−0.65	0.76	0.45	−0.20^*^	−0.21^*^	−0.11	−0.11	−0.15^*^	−0.21^*^
Bravery	15.14±3.82	−0.51	−0.66	0.76	0.44	−0.15^*^	−0.16^*^	−0.03	−0.07	−0.15^*^	−0.12
Creativity	14.01±4.17	−0.23	−0.90	0.80	0.50	−0.13	−0.19^*^	−0.06	−0.01	﻿﻿−0.14^*^	﻿−0.12
Curiosity	14.85±3.81	−0.42	−0.69	0.74	0.41	−0.09	−0.16^*^	−0.02	−0.03	−0.11	−0.09
Fairness	15.25±3.90	−0.51	−0.70	0.77	0.45	−0.22^*^	−0.17^*^	−0.10	−0.17^*^	−0.15^*^	−0.19^*^
Forgiveness	11.75±3.01	−0.57	−0.83	0.80	0.58	−0.19^*^	−0.13	−0.13	−0.11	−0.11	−0.13
Gratitude	16.73±3.13	−0.82	−0.08	0.62	0.30	−0.21^*^	−0.19^*^	−0.09	−0.13	−0.09	−0.18^*^
Honesty	11.26±3.12	−0.45	−0.88	0.76	0.53	−0.27^*^	−0.20^*^	−0.10	−0.18^*^	−0.12	−0.21^*^
Hope	15.41±3.74	−0.63	−0.43	0.72	0.39	−0.14^*^	−0.14^*^	−0.01	−0.06	−0.11	−0.11
Humility	12.96±3.64	0.04	−0.61	0.52	0.21	−0.18^*^	−0.21^*^	−0.05	−0.18^*^	−0.08	−0.20^*^
Humor	14.37±4.38	−0.36	−0.92	0.77	0.57	−0.13	−0.15^*^	−0.06	−0.07	−0.16^*^	−0.13
Judgment	14.21±4.18	−0.29	−0.79	0.82	0.53	−0.22^*^	−0.24^*^	−0.08	−0.11	−0.10	−0.19^*^
Kindness	14.41±4.53	−0.32	−0.58	0.66	0.34	−0.14^*^	−0.09	−0.02	−0.10	−0.07	−0.10
Leadership	12.17±4.34	0.13	−0.86	0.82	0.52	−0.21^*^	−0.25^*^	−0.07	−0.15^*^	−0.18^*^	−0.21^*^
Love	14.75±3.82	−0.48	−0.54	0.71	0.37	−0.24^*^	−0.21^*^	−0.16^*^	−0.16^*^	−0.17^*^	−0.21^*^
Love of learning	15.03±4.06	−0.48	−0.81	0.84	0.56	−0.13	−0.19^*^	−0.05	−0.05	−0.10	−0.12
Perseverance	14.68±3.81	−0.44	−0.51	0.78	0.47	−0.22^*^	−0.28^*^	−0.14^*^	−0.14^*^	−0.14^*^	−0.22^*^
Perspective	13.68±3.95	−0.13	−0.84	0.78	0.46	−0.19^*^	−0.24^*^	−0.14^*^	−0.12	−0.18^*^	−0.19^*^
Prudence	10.86±3.11	−0.36	−0.70	0.76	0.51	−0.20^*^	−0.23^*^	−0.08	−0.12	−0.11	−0.19^*^
Self-regulation	9.76±2.99	−0.12	−0.69	0.58	0.31	−0.30^*^	﻿−0.27^*^	−0.10	−0.23^*^	−0.13	−0.28^*^
Social intelligence	14.03±3.63	−0.16	−0.79	0.67	0.34	−0.19^*^	−0.19^*^	−0.12	−0.14^*^	−0.16^*^	−0.18^*^
Spirituality	13.55±3.81	−0.15	−0.79	0.69	0.35	−0.13^*^	−0.18^*^	−0.07	−0.09	−0.13	−0.14^*^
Zest	15.1±3.75	−0.35	−0.89	0.75	0.42	−0.22^*^	−0.20^*^	−0.17^*^	−0.14^*^	−0.21^*^	−0.18^*^
Teamwork	15.83±3.66	−0.64	−0.48	0.79	0.47	−0.20^*^	−0.18^*^	−0.11	−0.12	−0.13	−0.17^*^

*M* mean, *SD* standard deviation, 𝜶 Cronbach’s alpha coefficient, *N* = 521

^*^p < 0.002 (0.05/24 Bonferroni corrected)

### Comparisons of character strengths according to demographic variables

According to maternal education level, a significant difference was found in kindness (*F*_3, 300_ = 5.80, *p* = 0.001, *Cohen’s f* = 0.24). According to paternal education level, a significant difference was found in leadership (*F*_3, 300_ = 5.30, *p* = 0.001, *Cohen’s f* = 0.23). Post hoc tests showed that children whose parents have gone to college scored higher on those character strengths than those whose parents have a lower educational level. No significant group differences between gender, grade, only-child, left-behind experiences, residence, and family structure were found.

### Comparisons of behavior problems according to demographic variables

Girls showed significantly lower scores than boys in learning problem (*t*_519_ = 2.97, *p* = 0.003, *d* = 0.26). Students living in rural had higher scores in conduct problem than those living in urban (*t*_513_ = 2.95, *p* = 0.003, *d* = 0.26). And students with left-behind experiment showed higher anxiety scores than those who without (*t*_302_ = 2.76, *p* = 0.006, *d* = 0.34). According to maternal education level, a significant group difference was found in anxiety (*F*_3, 300_ = 4.16, *p* = 0.007, *Cohen’s f* = 0.2), and students whose mother with an education level of “primary or lower” scored higher than those with higher maternal education level. No significant group among the grade, only-child, paternal education level, and family structure were found.

### Correlations of the character strengths with the behavior problems

As shown in Table 2, all character strengths were negatively correlated with behavior problems (*r* = −0.14 to −0.3, *p* < 0.05 Bonferroni corrected). Those results were consistent with our assumption that higher-level character strength was associated with fewer behavior problems.

### Stepwise linear regression analysis between demographic factors and behavior problems

As shown in Table 3, three demographic factors, the residence, left-behind experiences, and maternal education level, showed independent contributions to behavior problems.

**Table 3:** Stepwise linear regression analysis between the dependent variable behavior problems and independent variable demographic factors

Dependent variable	Independent variable	Adjusted *R*^2^	*β*	Std. error	*t*	*p*
Conduct problem		4%				
	Residence		−0.14	0.05	−2.97	0.003
	Left-behind experiences		−0.10	0.05	−2.10	0.037
*F*		6.56				0.002
Learning problem		2%				
	Residence		−0.16	0.07	−2.46	0.015
*F*		6.04				0.015
Psychosomatic		2%				
	Left-behind experiences		−0.08	0.04	−2.39	0.018
*F*		5.70				0.018
Impulsive-hyperactive		1%				
	Residence		−0.15	0.07	−2.31	0.021
*F*		5.38				0.021
Anxiety		4%				
	Left-behind experiences		−0.15	0.06	−2.73	0.007
	Maternal education level		−0.08	0.03	﻿−2.70	0.007
*F*	7.91					< 0.001
Hyperactive index		2%				
	Left-behind experiences		−0.12	0.06	−2.16	0.032
	Residence		−0.12	0.06	−2.15	0.033
*F*		4.58				0.011

Only variables that showed independent contributions were presented, *p* < 0.05

### Findings related to the character strengths predicting behavior problems

Importantly, after accounting for the effect of demographic factors (residence, left-behind experiences, and maternal education level), character strengths additionally explained a fairly big proportion (14–22%) of the variances in various behavior problems. Particularly, character strengths additionally explained 22% of the variance (*F*_change_ [24, 276] = 3.5, *p* < 0.001) in conduct problem, 19% of the variance (*F*_change_ [24, 276] = 2.89, *p* < 0.001) in learning problem, 14% of the variance (*F*_change_ [24, 276] = 1.87, *p* = 0.01) in psychosomatic problem, 18% of the variance (*F*_change_ [24, 276] = 2.62, *p* < 0.001) in impulsive-hyperactive problem, and 19% of the variance (*F*_change_ [24, 276] = 2.76, *p* < 0.001) in hyperactive index (Table 4). Several character strengths, namely self-regulation, leadership, humility, zest, and perseverance, showed independent contributions in the models. Unexpectedly, however, hope seems to be promoting several behavior problems. The results were not confounded by multicollinearity as all tolerance values were bigger than 0.10 and VIF were less than 10 (≤ 5.63) (Hair et al., 1998).

**Table 4:** The final model of the variance in behavior problems explained by character strengths after controlling demographic factors

		Δ*R*^2^	*β*	*t*	*p*	VIF
Conduct problem	**Step1: Demographic factors**	0.04^*^				
	**Step2: Character strengths**	0.22^***^				
	Leadership		−0.20	−2.14	0.033	3.00
	Self-regulation		−0.31	−3.86	< 0.001	2.38
	**Summary**					
	*R*^2^	0.27				
	Adjust *R*^2^	0.19				
Learning problem	**Step1: Demographic factors**	0.03^*^				
	**Step2: Character strengths**	0.19^***^				
	Perseverance		−0.28	−2.40	0.017	4.86
	Self-regulation		−0.21	−2.52	0.012	2.38
	**Summary**					
	*R*^2^	0.23				
	Adjust *R*^2^	0.15				
Psychosomatic	**Step1: Demographic factors**	0.02				
	**Step2: Character strengths**	0.14^*^				
	Hope		0.34	3.30	0.001	3.55
	Zest		−0.23	−2.20	0.029	3.63
	**Summary**					
	*R*^2^	0.16				
	Adjust *R*^2^	0.08				
Impulsive-hyperactive	**Step1: Demographic factors**	0.03^*^				
	**Step2: Character strengths**	0.18^***^				
	Hope		0.21	2.04	0.042	3.55
	Humility		−0.14	−2.00	0.045	1.70
	Leadership		−0.19	−2.07	0.039	3.00
	Self-regulation		−0.33	−4.03	< 0.001	2.38
	**Summary**					
	*R*^2^	0.21				
	Adjust *R*^2^	0.13				
Hyperactive index	**Step1: Demographic factors**	0.04^*^				
	**Step2: Character strengths**	0.19^***^				
	Hope		0.22	2.24	0.026	3.55
	Self-regulation		−0.34	−4.11	< 0.001	2.38
	**Summary**					
	*R*^2^	0.22				
	Adjust *R*^2^	0.15				

*VIF* variance inflation factors

^*^p < 0.05, ^***^p < 0.001 in *F* change statistics. Only variables that showed independent contributions were presented, *p* < 0.05

## Discussion

This study revealed negative associations between character strengths and specific behavior problems among early adolescents. Stepwise linear regression showed that residence, left-behind experiences, and maternal education level had an independent contribution to behavior problems. Hierarchical regression analyses showed that character strengths explained a significant proportion of additional variance (14–22%) in behavior problems, after the effect of demographic factors was controlled. Several character strengths (i.e., self-regulation, leadership, humility, zest, and perseverance) were independently related to fewer behavior problems in early adolescents.

We found that the character strength of kindness was influenced by the maternal educational level and leadership was influenced by the paternal educational level. These results were in line with the previous findings that a higher maternal education level was positively associated with higher levels of character strengths (Reeves et al., 2014) and wellbeing (Mather & Foxen, 2010) and could exert a protective effect on children’s mental health (Lawrence et al., 2020). Moreover, the link of paternal educational level and leadership maybe account for by the positive association of paternal educational level with overall school achievement (Cornelius-White et al., 2016) and better communication skills of students (Umasyah & Alfiasari, 2016).

Our stepwise regressions found that residence, left-behind experience, and maternal educational level were associated with more behavior problems in adolescents. Consistent with previous findings (Li et al., 2013; Qu et al., 2008; Yu, 2010), this study found that left-behind experience was linked to more severe psychosomatic problems and conduct problems. Connectedness to parents had a positive and a governing effect on the development of children, which could promote children’s prosocial behaviors and prevent internalizing and externalizing problem behaviors (Day & Padilla-Walker, 2009). Children grownup with an insufficient company of parents thus could be prone to behavioral problems. In addition, this study found that the residence of children was related to learning problems and impulsive-hyperactive problems. These results were in line with previous findings among Chinese adolescents (Liu et al., 2009; Tao et al., 2007). In China, students who live in rural areas can access fewer educational resources and tend to have less academic achievement than urban students (Zhao & Bodovski, 2020). Also, students of the rural area could be more prone to hyperactivity than those in urban, given that ADHD prevalence estimates in rural areas were significantly higher than in urban areas (Liu et al., 2018). These factors could contribute to more severe behavior problems in the rural. The finding of maternal educational level associated with more behavior problems in children was in agreement with previous findings that maternal educational level exerted a protective effect on children’s mental health (Lawrence et al., 2020) and that parental education continued to be the strongest risk factor for parent-reported child mental health problems (e.g., emotional symptoms, conduct problems, and hyperactivity/inattention, as measured by the Strengths & Difficulties Questionnaire), with ORs increasing as parental educational level descended (Sonego et al., 2013).

There has been an increasing interest in character strengths that grooves a variety of positive psychology applications since the first inception of the conceptualization in applied psychology (Niemiec, 2013). To date, traditional models have heavily relied on disease models in promoting mental health and human potential that raising healthy, happy, and morally competent youths appear to be highly associated with promoting the strengths of character (Park & Peterson, 2008). The current study revealed that character strengths were negatively correlated with each behavior problem in early adolescence, which was consistent with previous findings in adolescents (Demirci et al., 2019; Shoshani & Shwartz, 2018) and adults (Casali et al., 2021; Cheng et al., 2020; Duan & Bu, 2019; Sueki, 2021). These negative correlations between character strengths and behavior problems suggest that, similar to adults, character strengths are associated with less severe behavior problems in early adolescents. However, as character strength traits are interrelated, at least some of these associations could be mediated by other traits or demographic variables. We hence further examined these relationships using hierarchical regression analyses. With the regression models, we analyzed the extent that character strengths were predictive of fewer behavior problems after controlling demographic factors, and explored specifically what traits contributed to which behavior problems.

The hierarchical regressions suggested that character strengths explained a significant proportion of variances in various behavior problems after controlling the effects of demographic factors. Moreover, character strengths including self-regulation, leadership, humility, zest, and perseverance had independent contributions to behavior problems. These results were in line with the findings of Demirci et al. (2019) and Shoshani and Shwartz (2018) suggesting that character strengths could be important protective factors in the positive development of youth that buffers against mental health difficulties.

In extending previous findings, our results identified specific character strengths, i.e., self-regulation, humility, leadership, perseverance, and zest, that were related to specific behavior problems. Self-regulation was independently contributed to less conduct, impulsive-hyperactive, learning problems, and lower hyperactive index. Self-regulation is the ability to control one’s impulsive feelings, thoughts, and behavior to comply with social and personal standards to achieve long-term goals (Moffitt et al., 2011). A higher level of self-regulation thus could be crucial in reducing inappropriate behaviors in children. A study found that low self-regulation during childhood was predictive of an increased risk of smoking throughout adulthood (Daly et al., 2016). Humility involves holding a real, secure, and open view of themselves as well as an appreciation of the value and contribution of others (Chancellor & Lyubomirsky, 2013), which plays an important role in repairing and forming relationships with strong social bonds (Davis et al., 2013), and is associated with greater personal wellbeing (Krause et al., 2016) and better resilience from anxiety (Kesebir, 2014). In the current study, humility was protective of fewer learning problems, suggesting that humble children might enjoy a more secure and open mind toward learning. The trait of leadership was predictive of less conduct and impulsive-hyperactive problem in this study. Leadership was associated with positive self-perceptions in various domains, suggesting that a child with high leadership hence could have better control of their own behaviors (Scharf & Mayseless, 2009). Perseverance was linked to fewer learning problems in our results. Perseverance is conceptually aligned with the industriousness aspect of the Big Five personality trait conscientiousness (DeYoung et al., 2007), and the latter was found as a strong predictor of academic achievement, lower levels of negative affect (Fayard et al., 2012). Finally, zest was linked to fewer psychosomatic problems. Zest means approaching a situation, or life in general, with excitement and energy (VIA Institute on Character, 2022). Zest was proposed to indicate an overall positive mental health state (Petkari & Ortiz-Tallo, 2018). The effect of zest hence could be accounted for by a more active attitude toward life experiences.

Unexpectedly, this study found that hope was predictive of more psychosomatic and impulsive-hyperactive problems. Positive psychology has a strong propensity to promote positive experiences and human strengths. However, it is not necessarily implying that character strength has only positive effects on every aspect of development. Peterson & Seligman (2006) proposed that deviations from human strengths in terms of under, over, and opposite expression would be predictive of counterproductive outcomes and maybe psychopathology. For example, the overuse of social intelligence and humility was associated with social anxiety (Freidlin et al., 2017). Our results hence suggest that overexpression of certain character strengths could lead to counterproductive outcomes.

This study has several limitations that deserve consideration. Firstly, this study used a regional sample that restricts to students of two cities in China. Although the character strengths were proposed to be ubiquitous and perhaps universal across cultures (Park et al., 2006), cautions need to be taken when applying these conclusions to a sample of other regions. Secondly, the current study focused on the association between character strengths and behavior problems, leaving other factors that showed an impact on behavior problems, such as the experience of physical and emotional abuse (Cui & Liu, 2020), unattained. Thirdly, the present study adopted a cross-sectional design which precludes causal inference. Future prospective studies on potential causal relationships between character strengths and behavior problems (i.e., if adolescents with high character strengths are less likely to suffer behavior problems) are needed. Finally, the sample size of the current study was relatively small, which could limit the power to detect more subtle effects. Despite these limitations, the study has some strong points. Firstly, to the best of our knowledge, this is the first study that revealed links between character strengths and specific behavior problems in early adolescents, and these links exist in children of various demographic settings. Secondly, this study adopted commonly used measures of character strengths and behavior problems (VIA-Youth and PSQ); our results hence can be easily compared with other studies on this topic.

## Conclusions

The current study revealed that character strengths were independently and negatively associated with behavior problems, suggesting that character strengths could be a protective factor against behavior problems in early adolescents. These findings could shed light on the prevention and intervention of behavior problems in several ways. For instance, character strengths are, overall, associated with fewer behavior problems, suggesting that cultivating character strengths in children would be generally beneficial and thus should be encouraged. Furthermore, when considering the priority in cultivating character strengths, self-regulation, perseverance, zest, humility, and leadership should be highlighted as they were independently and significantly related to fewer behavior problems. Finally, children of rural areas, with left-behind experiences, and with low maternal educational levels may be most beneficial from character strength training, as they tend to have more behavior problems.

## Supplementary Information

**Additional file 1: Table S1.** Sample Variables and Behavior Problems. **Table S2.** Descriptive statistics, and Pearson correlations between the 24 character strengths and behavior problems. **Table S3.** Stepwise linear regression analysis between the dependent variable behavior problems and independent variable demographic factors. **Table S4.** The final model of the variance in behavior problems explained by character strengths after controlling demographic factors.

## Data Availability

The datasets used and/or analyzed during the current study are available from the corresponding authors on reasonable request.
