# Supplementary material for: Character strengths as protective factors against behavior problems in early adolescent
Source: Psicol Reflex Crit. 2022 Jun 1;35:16. doi: 10.1186/s41155-022-00217-z (PMC9156651; doi:10.1186/s41155-022-00217-z)
Supplement: Supplementary file 1 — Additional file 1: Table S1. Sample Variables and Behavior Problems. Table S2. Descriptive statistics, and Pearson correlations between the 24 character strengths and behavior problems. Table S3. Stepwise linear regression analysis between the dependent variable behavior problems and independent variable demographic factors. Table S4. The final model of the variance in behavior problems explained by character strengths after controlling demographic factors. [file 41155_2022_217_MOESM1_ESM.docx]

Table S1. Sample Variables and Behavior Problems

| Variables | Options | n (%) | M ±SD |
| --- | --- | --- | --- |
| Gender (boy vs. girl) | Boy | 253(47.9) |  |
| Age (years) | 10 | 189(35.8) |  |
|  | 11 | 185(35) |  |
|  | 12 | 147(27.8) |  |
|  | 13 | 7(1.3) |  |
| Grade | 4 | 168(31.8) |  |
|  | 5 | 150(28.4) |  |
|  | 6 | 210(39.8) |  |
| Resident (urban vs. rural) | Urban | 313(60) |  |
| Only-child or not | Only-child | 215(41.2) |  |
| Left-behind experience | Yes | 124(40) |  |
| Family structure | Two-parent family | 135(43.5) |  |
|  | Multigenerational family | 119(38.4) |  |
|  | Other | 56(18.1) |  |
| Maternal education level | Primary or lower | 48(15.5) |  |
|  | Junior middle school | 128(41.3) |  |
|  | Senior middle school | 71(22.9) |  |
|  | College or higher | 63(20.3) |  |
| Paternal education level | Primary or lower | 49(15.8) |  |
|  | Junior middle school | 136(43.9) |  |
|  | Senior middle school | 73(23.5) |  |
|  | College or higher | 52(16.8) |  |
| Behavior problem | Conduct problems |  | 0.43 ± 0.39 |
|  | Learning problems |  | 0.73 ± 0.58 |
|  | Psychosomatic |  | 0.18 ± 0.30 |
|  | Impulsivity-hyperactivity |  | 0.53 ± 0.54 |
|  | Anxiety |  | 0.48 ± 0.46 |
|  | Hyperactivity index |  | 0.53 ± 0.45 |

Table S2. Descriptive statistics, and Pearson correlations between the 24 character strengths and behavior problems

|  | M±SD | Skewness | Kurtosis | 𝜶 | Conduce problem | Learning problem | Psycho-somatic | Impulsive-hyperactive | Anxiety | Hyperac-  tive index |
| --- | --- | --- | --- | --- | --- | --- | --- | --- | --- | --- |
| Appreciation of Beauty & Excellence | 15.39±3.98 | -0.56 | -0.66 | 0.77 | -0.20^*^ | -0.21^*^ | -0.1 | -0.11 | -0.15^*^ | -0.21^*^ |
| Bravery | 15.14±3.82 | -0.5 | -0.68 | 0.77 | -0.15^*^ | -0.17^*^ | -0.03 | -0.07 | -0.16^*^ | -0.12 |
| Creativity | 14.01±4.16 | -0.23 | -0.89 | 0.81 | -0.13 | -0.19^*^ | -0.05 | -0.01 | -0.14 | -0.12 |
| Curiosity | 14.85±3.81 | -0.41 | -0.7 | 0.75 | -0.09 | -0.17^*^ | -0.02 | -0.03 | -0.11 | -0.09 |
| Fairness | 15.26±3.9 | -0.5 | -0.71 | 0.71 | -0.23^*^ | -0.17^*^ | -0.1 | -0.17^*^ | -0.16^*^ | -0.19^*^ |
| Forgiveness | 11.75±3.01 | -0.56 | -0.82 | 0.8 | -0.20^*^ | -0.14^*^ | -0.13^*^ | -0.12 | -0.12 | -0.14^*^ |
| Gratitude | 16.74±3.13 | -0.82 | -0.09 | 0.63 | -0.20^*^ | -0.19^*^ | -0.08 | -0.13^*^ | -0.08 | -0.18^*^ |
| Honesty | 11.28±3.12 | -0.45 | -0.88 | 0.78 | -0.26^*^ | -0.21^*^ | -0.09 | -0.18^*^ | -0.12 | -0.21^*^ |
| Hope | 15.41±3.74 | -0.62 | -0.43 | 0.73 | -0.14^*^ | -0.15^*^ | -0.01 | -0.06 | -0.11 | -0.11 |
| Humility | 12.96±3.64 | 0.04 | -0.62 | 0.53 | -0.19^*^ | -0.22^*^ | -0.05 | -0.18^*^ | -0.09 | -0.20^*^ |
| Humor | 14.36±4.37 | -0.36 | -0.91 | 0.85 | -0.12 | -0.15^*^ | -0.06 | -0.07 | -0.15^*^ | -0.12 |
| Judgment | 14.21±4.17 | -0.29 | -0.79 | 0.85 | -0.22^*^ | -0.24^*^ | -0.07 | -0.11 | -0.1 | -0.19^*^ |
| Kindness | 14.41±3.52 | -0.32 | -0.57 | 0.68 | -0.13 | -0.08 | -0.01 | -0.1 | -0.06 | -0.1 |
| Leadership | 12.18±4.35 | 0.13 | -0.87 | 0.81 | -0.20^*^ | -0.25^*^ | -0.07 | -0.15^*^ | -0.17^*^ | -0.20^*^ |
| Love | 14.74±3.83 | -0.47 | -0.56 | 0.72 | -0.24^*^ | -0.22^*^ | -0.16^*^ | -0.16^*^ | -0.17^*^ | -0.21^*^ |
| Love of Learning | 15.04±4.07 | -0.47 | -0.83 | 0.85 | -0.14 | -0.20^*^ | -0.05 | -0.06 | -0.1 | -0.13 |
| Perseverance | 14.67±3.81 | -0.44 | -0.53 | 0.79 | -0.22^*^ | -0.28^*^ | -0.13 | -0.14^*^ | -0.14^*^ | -0.22^*^ |
| Perspective | 13.7±3.95 | -0.14 | -0.85 | 0.79 | -0.19^*^ | -0.24^*^ | -0.13 | -0.11 | -0.18^*^ | -0.19^*^ |
| Prudence | 10.87±3.11 | -0.36 | -0.7 | 0.76 | -0.20^*^ | -0.23^*^ | -0.07 | -0.11 | -0.19^*^ | -0.20^*^ |
| Self-Regulation | 9.76±2.98 | -0.12 | -0.69 | 0.57 | -0.29^*^ | -0.27^*^ | -0.1 | -0.23^*^ | -0.12^*^ | -0.28^*^ |
| Social Intelligence | 14.03±3.64 | -0.16 | -0.79 | 0.7 | -0.18^*^ | -0.19^*^ | -0.11 | -0.13 | -0.15^*^ | -0.18^*^ |
| Spirituality | 13.52±3.81 | -0.14 | -0.8 | 0.69 | -0.13 | -0.18^*^ | -0.08 | -0.08 | -0.13 | -0.14 |
| Zest | 15.1±3.75 | -0.35 | -0.89 | 0.79 | -0.21^*^ | -0.20^*^ | -0.16^*^ | -0.13 | -0.20^*^ | -0.18^*^ |
| Teamwork | 15.83±3.66 | -0.63 | -0.5 | 0.76 | -0.20^*^ | -0.18^*^ | -0.11 | -0.13 | -0.13 | -0.18^*^ |

Note. M = mean, SD = standard deviation, 𝜶 = Cronbach’s alpha coefficient, N = 528. ^*^p < 0.002 (0.05/24 Bonferroni corrected).

Table S3. Stepwise linear regression analysis between the dependent variable behavior problems and independent variable demographic factors

| Dependent variable | Independent variable | Adjusted R^2^ | β | Std. error | t | p |
| --- | --- | --- | --- | --- | --- | --- |
| Conduct problem |  | 4% |  |  |  |  |
|  | Residence |  | -0.14 | 0.05 | -2.88 | 0.004 |
|  | Left-behind experiences |  | -0.11 | 0.05 | -2.29 | 0.022 |
| F |  | 6.84 |  |  |  | 0.001 |
| Learning problem |  | 2% |  |  |  |  |
|  | Residence |  | -0.16 | 0.07 | -2.40 | 0.017 |
|  | Left-behind experiences |  | -0.13 | 0.07 | -1.99 | 0.048 |
| F |  | 4.88 |  |  |  | 0.008 |
| Psychosomatic |  | 3% |  |  |  |  |
|  | Left-behind experiences |  | -0.09 | 0.04 | -2.48 | 0.014 |
|  | Paternal education level |  | -0.04 | 0.02 | -2.01 | 0.045 |
| F |  | 5.53 |  |  |  | 0.004 |
| Impulsive-hyperactive |  | 1% |  |  |  |  |
|  | Residence |  | -0.14 | 0.07 | -2.17 | 0.031 |
| F |  | 4.69 |  |  |  | 0.031 |
| Anxiety |  | 5% |  |  |  |  |
|  | Left-behind experiences |  | -0.16 | 0.06 | -2.98 | 0.003 |
|  | Maternal education level | | -0.08 | 0.03 | -2.87 | 0.004 |
| F | 9.33 | |  |  |  | < 0.001 |
| Hyperactive index | | 2% |  |  |  |  |
|  | Left-behind experiences |  | -0.13 | 0.06 | -2.25 | 0.025 |
|  | Residence |  | -0.11 | 0.05 | -2.04 | 0.042 |
| F |  | 4.63 |  |  |  | 0.01 |

Only variables that showed independent contributions were presented, p < 0.05.

Table S4. The final model of the variance in behavior problems explained by character strengths after controlling demographic factors

|  |  | ΔR^2^ | β | t | p | VIF |
| --- | --- | --- | --- | --- | --- | --- |
| Conduct problem | **Step1: Demographic factors** | 0.04^*^ |  |  |  |  |
|  | **Step2: Character strengths** | 0.22^***^ |  |  |  |  |
|  | leadership |  | -0.19 | -2.09 | 0.037 | 3.04 |
|  | perspective |  | 0.25 | 2.02 | 0.044 | 5.71 |
|  | self-regulation |  | -0.31 | -3.96 | < 0.001 | 2.34 |
|  | **Summary** |  |  |  |  |  |
|  | R^2^ | 0.27 |  |  |  |  |
|  | Adjust R^2^ | 0.19 |  |  |  |  |
| Learning problem | **Step1: Demographic factors** | 0.03^*^ |  |  |  |  |
|  | **Step2: Character strengths** | 0.20^***^ |  |  |  |  |
|  | humility |  | -0.14 | -2.03 | 0.043 | 1.71 |
|  | perseverance |  | -0.28 | -2.44 | 0.015 | 4.91 |
|  | self-regulation | | -0.22 | -2.71 | 0.007 | 2.34 |
|  | **Summary** |  |  |  |  |  |
|  | R^2^ | 0.23 |  |  |  |  |
|  | Adjust R^2^ | 0.15 |  |  |  |  |
| Psychosomatic | **Step1: Demographic factors** | 0.04^*^ |  |  |  |  |
|  | **Step2: Character strengths** | 0.14^*^ |  |  |  |  |
|  | hope |  | 0.35 | 3.37 | 0.001 | 3.56 |
|  | perseverance |  | -0.28 | -2.34 | 0.02 | 4.91 |
|  | **Summary** |  |  |  |  |  |
|  | R^2^ | 0.17 |  |  |  |  |
|  | Adjust R^2^ | 0.09 |  |  |  |  |
| Impulsive-hyperactive | **Step1: Demographic factors** | 0.03 |  |  |  |  |
|  | **Step2: Character strengths** | 0.18^***^ |  |  |  |  |
|  | humility |  | -0.15 | -2.20 | 0.028 | 1.71 |
|  | self-regulation |  | -0.36 | -4.39 | < 0.001 | 2.34 |
|  | **Summary** |  |  |  |  |  |
|  | R^2^ | 0.21 |  |  |  |  |
|  | Adjust R^2^ | 0.13 |  |  |  |  |
| Hyperactive index | **Step1: Demographic factors** | 0.04^*^ |  |  |  |  |
|  | **Step2: Character strengths** | 0.19^***^ |  |  |  |  |
|  | hope |  | 0.22 | 2.20 | 0.029 | 3.56 |
|  | self-regulation |  | -0.07 | -7.21 | < 0.001 | 2.05 |
|  | **Summary** |  |  |  |  |  |
|  | R^2^ | 0.23 |  |  |  |  |
|  | Adjust R^2^ | 0.15 |  |  |  |  |

^*^ p < 0.05, ^***^ p < 0.001 in F change statistics. Only variables that showed independent contributions were presented, p < 0.05. VIF, variance inflation factors.
